# Supplementary material for: Migration dynamics of an important rice pest: The brown planthopper (Nilaparvata lugens) across Asia—Insights from population genomics
Source: Evol Appl. 2020 Jul 11;13(9):2449–59. doi: 10.1111/eva.13047 (PMC7513714; doi:10.1111/eva.13047)
Supplement: Supplementary file 1 — Supplementary Material [file EVA-13-2449-s001.pdf]

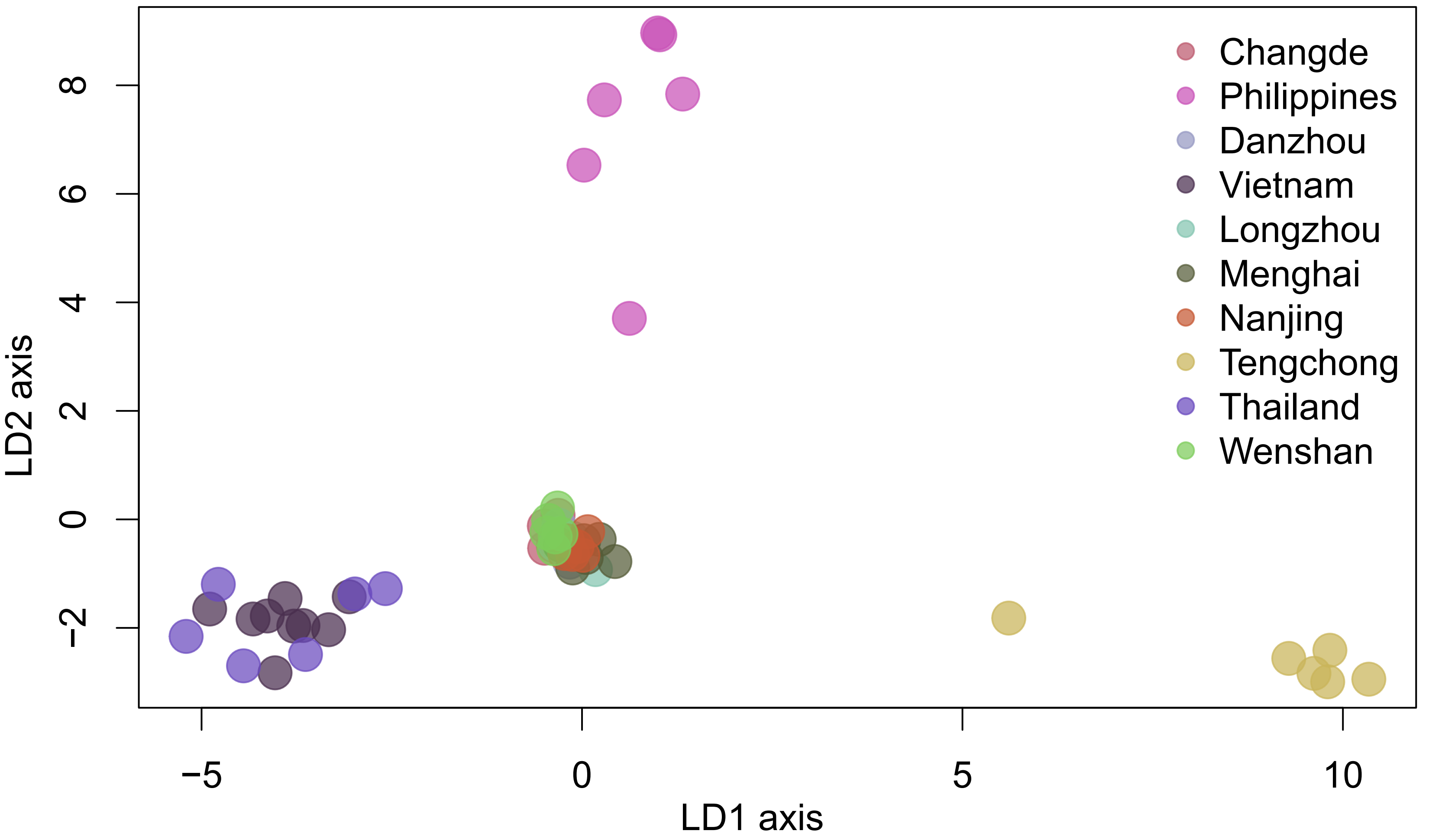

Supplementary Figure 1. Plot of the assignment of the northern populations to the three putative sources, Philippines (top centre), “Indochinese peninsular” (bottom left) and Tengchong (bottom right), using DAPC.
