# Supplementary material for: Migration dynamics of an important rice pest: The brown planthopper (Nilaparvata lugens) across Asia—Insights from population genomics
Source: Evol Appl. 2020 Jul 11;13(9):2449–59. doi: 10.1111/eva.13047 (PMC7513714; doi:10.1111/eva.13047)
Supplement: Supplementary file 4 — Supplementary Material [file EVA-13-2449-s004.pdf]

Supplementary Table 3. Pairwise F_{ST}'s calculated using Weir & Cockerham 1984 as implemented in hierfstat below the diagonal, with 95% bounds above the diagonal calculated following the bootstrapping procedure implemented in hierfstat with 1,000 replicates.

	Changde (China)	Los Banos (Philippines)	Danzhou (China)	Ho Chi Minh (Vietnam)	Longzhou (China)	Menghai (China)	Nanjing (China)	Tengchong (China)	Bangkok (Thailand)	Wenshan (China)
Changde		(0.0234 - 0.0242)	(0.001 - 0.0016)	(0.0072 - 0.0078)	(0.0021 - 0.0027)	(0.0039 - 0.0045)	(0.0067 - 0.0074)	(0.016 - 0.0167)	(0.0067 - 0.0074)	(0.0018 - 0.0024)
Philippines	0.0238		(0.0196 - 0.0204)	(0.0206 - 0.0213)	(0.022 - 0.0228)	(0.0211 - 0.0219)	(0.0305 - 0.0314)	(0.0324 - 0.0333)	(0.021 - 0.0218)	(0.0189 - 0.0196)
Danzhou	0.0013	0.0200		(0.0019 - 0.0025)	(0.0002 - 0.0008)	(0.0007 - 0.0012)	(0.0071 - 0.0077)	(0.0093 - 0.0099)	(0.0016 - 0.0021)	(0 - 0.0005)
Vietnam	0.0075	0.0210	0.0022		(0.002 - 0.0025)	(0.002 - 0.0025)	(0.0101 - 0.0107)	(0.0121 - 0.0127)	(0.0018 - 0.0023)	(0.0045 - 0.005)
Longzhou	0.0024	0.0224	0.0005	0.0023		(0.0014 - 0.002)	(0.005 - 0.0057)	(0.0094 - 0.01)	(0.003 - 0.0035)	(0.0021 - 0.0027)
Menghai	0.0041	0.0215	0.0009	0.0022	0.0017		(0.0086 - 0.0093)	(0.0069 - 0.0075)	(0.0016 - 0.0021)	(0.0019 - 0.0024)
Nanjing	0.0071	0.0309	0.0074	0.0104	0.0054	0.0089		(0.0182 - 0.0189)	(0.0117 - 0.0124)	(0.0083 - 0.009)
Tengchong	0.0163	0.0329	0.0096	0.0124	0.0097	0.0072	0.0186		(0.0117 - 0.0124)	(0.0134 - 0.0141)
Thailand	0.0070	0.0214	0.0018	0.0021	0.0032	0.0018	0.0121	0.0120		(0.0035 - 0.0041)
Wenshan	0.0021	0.0193	0.0002	0.0047	0.0024	0.0022	0.0086	0.0137	0.0038	
